# CT pulmonary angiography appropriateness in a single emergency department: does the use of revised Geneva score matter?

**DOI:** 10.1007/s11547-021-01416-x

**Published:** 2021-09-13

**Authors:** Alessandra Mirabile, Nicola Maria Lucarelli, Enza Pia Sollazzo, Amato Antonio Stabile Ianora, Angela Sardaro, Gianmario Mirabile, Filomenamila Lorusso, Vito Racanelli, Nicola Maggialetti, Arnaldo Scardapane

**Affiliations:** 1 Interdisciplinary Department of Medicine, Section of Diagnostic Imaging, University of Bari Medical School “Aldo Moro”, Piazza Giulio Cesare 11, 70124 Bari, Italy; 2 Department of Biomedical Sciences and Human Oncology, University of Bari Medical School “Aldo Moro”, Piazza Giulio Cesare 11, 70124 Bari, Italy; 3 Department of Electrical and Information Engineering, Polytechnic University of Bari, 70125 Bari, Italy; 4 Department of Basic Medical Sciences, Neuroscience and Sense Organs, (DSMBNOS), University of Bari Medical School “Aldo Moro”, Piazza Giulio Cesare 11, 70124 Bari, Italy

**Keywords:** Pulmonary embolism, Age-adjusted D-dimer cut-offs, Revised Geneva score, Computed tomography pulmonary angiography

## Abstract

**Purpose:**

To assess the percentage of computed tomography pulmonary angiography (CTPA) procedures that could have been avoided by methodical application of the Revised Geneva Score (RGS) coupled with age-adjusted D-dimer cut-offs rather than only clinical judgment in Emergency Department patients with suspected pulmonary embolism (PE).

**Material and methods:**

Between November 2019 and May 2020, 437 patients with suspected PE based on symptoms and D-dimer test were included in this study. All patients underwent to CTPA. For each patient, we retrospectively calculated the age-adjusted D-dimer cut-offs and the RGS in the original version. Finally, CT images were retrospectively reviewed, and the presence of PE was recorded.

**Results:**

In total, 43 (9.84%) CTPA could have been avoided by use of RGS coupled with age-adjusted D-dimer cut-offs. Prevalence of PE was 14.87%. From the analysis of 43 inappropriate CTPA, 24 (55.81%) of patients did not show any thoracic signs, two (4.65%) of patients had PE, and the remaining patients had alternative thoracic findings.

**Conclusion:**

The study showed good prevalence of PE diagnoses in our department using only physician assessment, although 9.84% CTPA could have been avoided by methodical application of RGS coupled with age-adjusted D-dimer cut-offs.

## Introduction

Acute pulmonary embolism (PE) represents a relatively common cardiopulmonary emergency; it consists of a partial or complete acute vessel obstruction, resulting in possible right ventricular failure. Deep vein thrombosis (DVT) is the complete or partial obstruction of one or more veins of the deep venous circle of the limbs and/or abdomen and pelvis. PE and DVT are two clinical entities of venous thromboembolism (VTE) and share the same predisposing factors [1, 2]. Epidemiological studies have shown that the incidence rates for PE range from 39 to 115 per 100.000 person years; for DVT, incidence rates range from 53 to 162 per 100.000 person years [3].

The clinical presentation of PE is often non-specific, so its diagnosis is difficult to achieve and can be misunderstood. The promptness of the diagnosis is essential since treatment improves prognosis. Computed tomography pulmonary angiography (CTPA) is the gold standard diagnostic technique to evaluate patients with a suspected PE [4].

The 2019 guidelines for the diagnosis and management of acute pulmonary embolism, developed by European Society of Cardiology in collaboration with the European Respiratory Society, provide important innovations including diagnostic algorithms for patients with suspected acute PE, with and without hemodynamic instability [5]. The guidelines suggest the risk stratification that can be based on either a physician’s unstructured estimate (i.e., “gestalt”) or a application of prediction rules, as Revised Geneva Score (RGS). Since PE post-test probability depends on the pre-test probability, it is essential that noninvasive diagnostic tests exclude PE in patients with a low pre-test clinical probability. D-dimer cut-off values adjusted for age rather than the fixed cut-off value can be a tool to increase the percentage of patients in whom PE could be excluded [6]. In many hospitals, different prediction rules are not systematically applied in favor of implicit physician’s estimate [7, 8].

The aims of this study were: (1) to determine the prevalence of acute PE in patients who underwent CTPA for suspected PE in our hospital; (2) to establish if the systematic application of the Revised Geneva Score (RGS) coupled with age-adjusted D-dimer cut-offs in the Emergency Department can lead to better selection of patients who undergo CTPA, rather than the empirical clinical judgment alone; (3) to identify in the patients with pre-test clinical probability “PE UNLIKELY” and D-dimer normal value, any other pulmonary radiological signs able to justify the onset of clinical symptoms.

## Methods

**Fig. 1:**
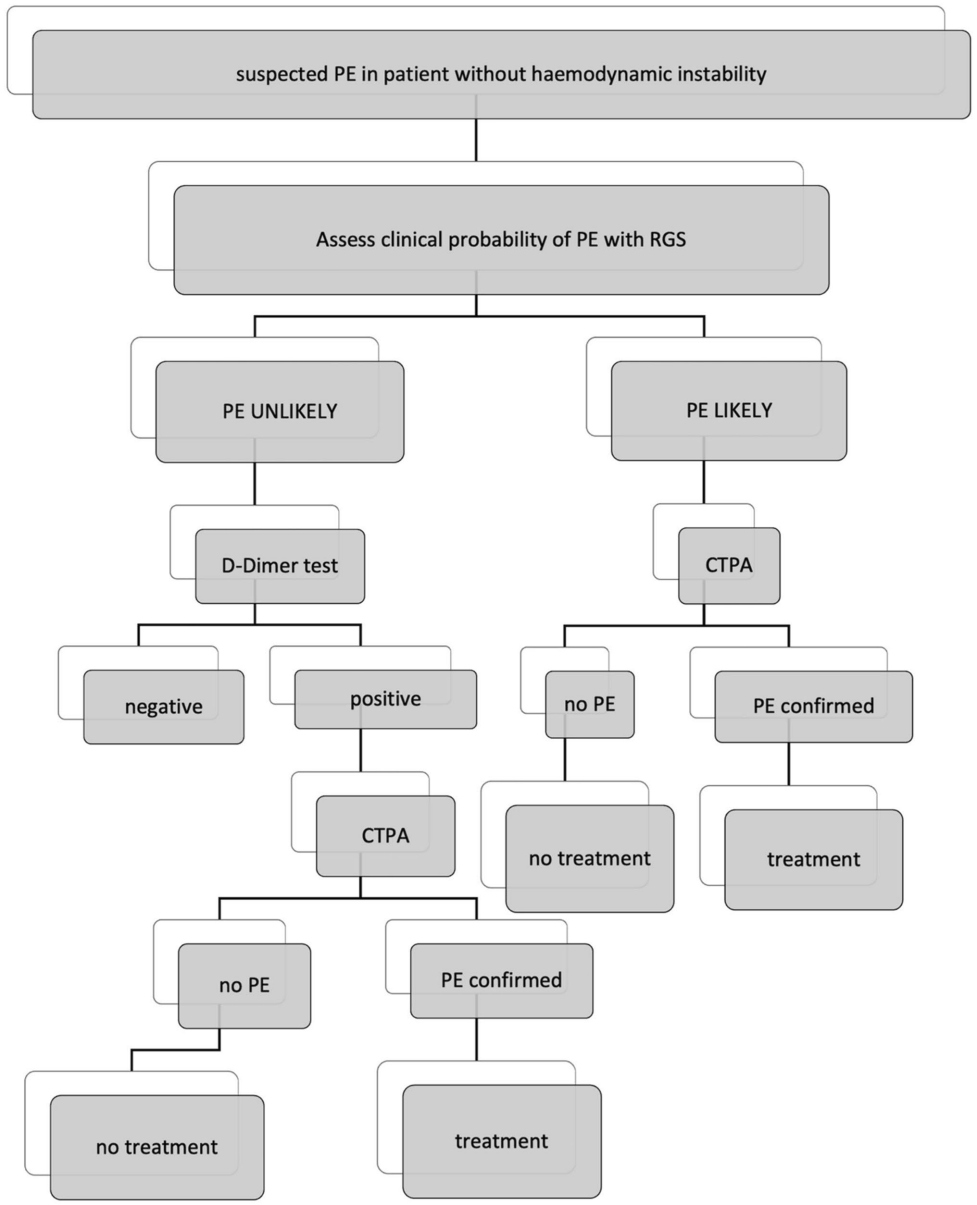
Diagnostic algorithm for patients with suspected pulmonary embolism without hemodynamic instability

All 475 pulmonary CTPA and clinical records of adult (> 18 years), hemodynamically stable, and SarsCov2 negative patients admitted to the emergency department of the university hospital Policlinico of Bari (Italy) for suspected pulmonary embolism (PE) in the period from November 2019 to May 2020 were retrieved from our RIS-PACS system. 38/475 (8%) CTPA were excluded from further evaluation as D-dimer test was not available, and consequently, 437 studies were retained. PE suspicion for all patients was based on symptoms and D-Dimer test (immunoturbidimetric method); no pre-test clinical probability was assessed at the moment of emergency admission. All CTPA exams were required by senior emergency physicians of our center with work experience ranging from 5 to 15 years. Patients were deemed as hemodynamically instable if one of the following three clinical manifestations was present: cardiac arrest, obstructive shock, and persistent hypotension. In order to establish the appropriateness of CTPA in the light of clinical probability, the RGS in the original version [9] was retrospectively calculated in all the cases. The RGS evaluates eight variables divided in: (a) predisposing risk factors (age over 65 years, DVT or PE previous episode, surgery under general anesthesia or fracture of lower limb in the last month, active cancer as solid or hematologic malignant active neoplasia or considered cured for less than 1 year), (b) symptoms (unilateral lower limb pain, hemoptysis), (c) clinical signs (heart rate values and pain on lower limb deep venous palpation and unilateral edema). One point is assigned for each of these variables; the sum of them lead to a score which identifies two groups of pre-test clinical probability (PE likely and PE unlikely) (Table 1). For each patient, we calculated the age-adjusted D-dimer cut-offs multiplying by 10 every decade of age over 50 years (age × 10 μg/L, for patients aged > 50 years). To assess CT appropriateness, the diagnostic algorithm suggested in recent guideline [10] (Fig. 1): CTPA was considered appropriate in patients with “PE LIKELY” category and in those with “PE UNLIKELY” category for D-Dimer levels, adjusted for age, increased.

**Table 1:** Revised Geneva Score, original version

Items	Points
Previous PE o DVT	3
Heart rate	
75–94 beats/min	3
≥ 95 beats/min	5
Surgery or fracture within the past month	2
Hemoptysis	2
Active cancer	2
Unilateral lower limb pain	3
Pain on lower limb deep venous palpation and unilateral edema	4
Age > 65 years	1
PE unlikely	**0–5**
PE likely	** ≥ 6**

Bold value indicates the sum of the RGS variables points which identifies two groups of pre-test clinical probability (PE likely/PE unlikely)

All CT exams were obtained with a 128 slices multi-detector CT (Siemens Somatom Definition DS). We performed an unenhanced scan in supine position from the jugule to the diaphragmatic domes, followed by a CTPA. CTPA was performed through the injection of 50 ml of iodinated contrast agent (Iomeprol 400 mgI/ml) followed by 40 ml of saline solution at a flow rate of 4 ml/s, into the cubital vein through a 16-18G needle, with the use of a bolus-tracking technique and a threshold of 100 HU in the main pulmonary artery. The scan delay was 6 s. Images were acquired in free breath using the following parameters: slice thickness 0.6 mm, tube voltage 100 kVp, rotation time 0.33 s, pitch 1.2, and acquisition time 2.94 s. Images were reconstructed with a slice thickness of 1 mm in mediastinal and parenchymal windows. The acquired data were subsequently transferred to our PACS workstation (Carestream Health, Rochester, NY). Axial, MultiPlanar Reformatting (MPR) and 3D Maximum Intensity Projection (3D MIP) images were retrospectively reviewed by two radiologists (A.S. and A.M., senior specialist consultant and fellow, respectively), and final decision was reached by consensual discussion. The presence of PE or any other pulmonary finding explaining symptoms was recorded.

## Results

Complete clinical records are given in Table 2. The patients were predominantly elderly (age mean 72 years) and male. The most common presenting symptoms were 176/437 (40.27%) dyspnea, followed by 51/437 (11.67%) syncope, 48/437 (10.98%) thoracalgia, 38/437 (8.7%) fever, 17/437 (3.89%) suggestive signs of DVT, 16/437 (3.66%) hemoptysis/hemoptoe, 10/437 (2.29%) cough and finally in an important percentage 81/437 (18,54%) a clinical onset with symptoms not exclusively PE suggestive (e.g., vomiting, abdominal pain, suspected concomitant cerebral ischemic stroke, altered state of consciousness, suspected venous thrombosis in districts other than lower limb, neoplastic fatigue, etc.).

**Table 2:** Clinical characteristics of patients with suspected PE

Characteristics of patients	*n*, %
Male	245 (56%)
Age mean	72 years
Age > 65 years	316 (72.31%)
*Clinical onset*	
Dyspnea	176 (40.27%)
Syncope	51 (11.67%)
Thoracalgia	48 (10.98%)
Fever	38 (8.7%)
Cough	10 (2.29%)
Hemoptysis/hemoptoe	16 (3.66%)
Suggestive signs of DVT	17 (3.89%)
Other clinical onset	81 (18.54%)
*Risk factors*	
Previous PE or DVT	25 (5.72%)
Immobilization within the past month	120 (27.46%)
Surgery or fracture within the past month	14 (3.2%)
Heart rate ≥ 95 beats/min	129 (29.52%)
Active cancer	65 (14.87%)

**Fig. 2:**
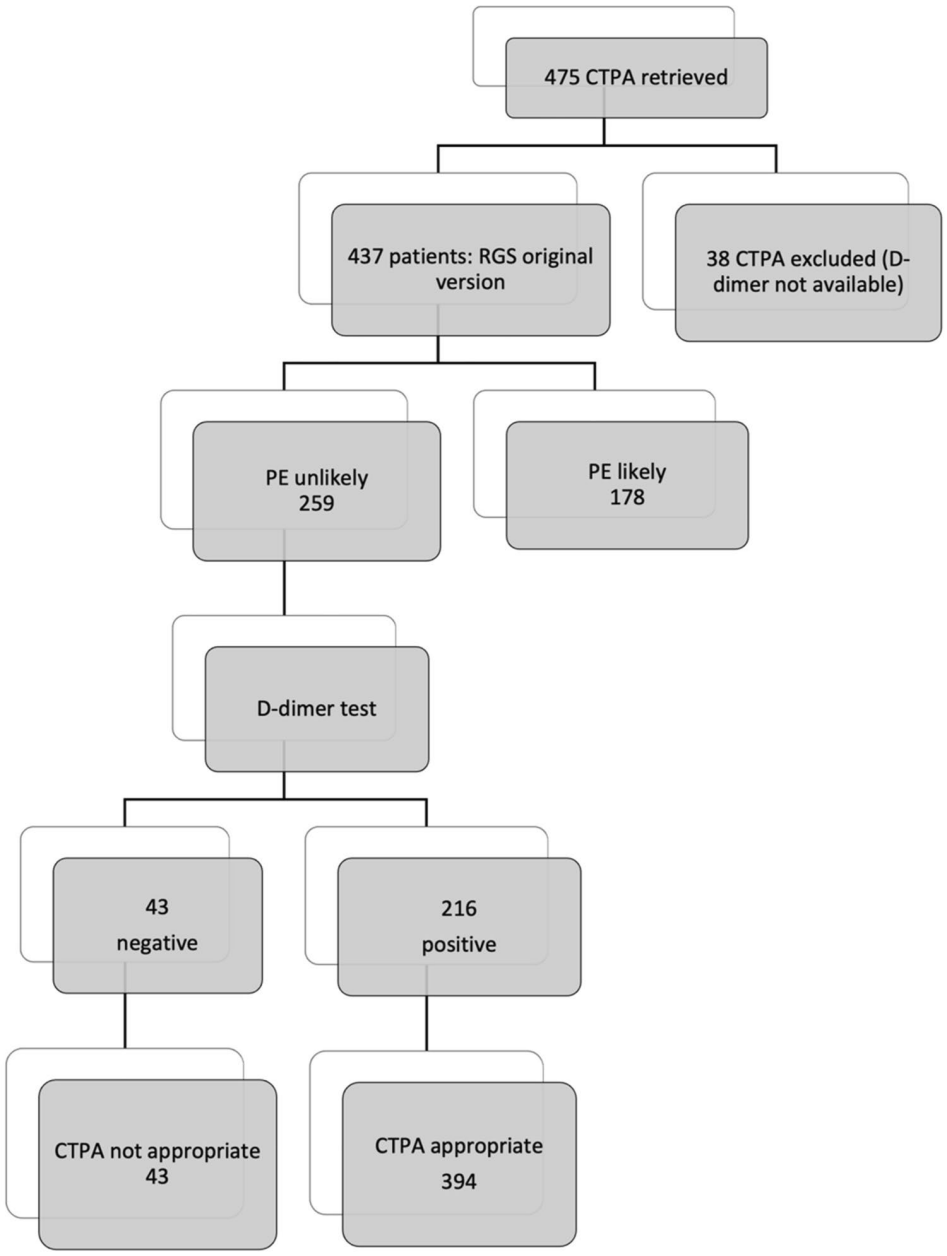
Study population flow diagram

**Fig. 3:**
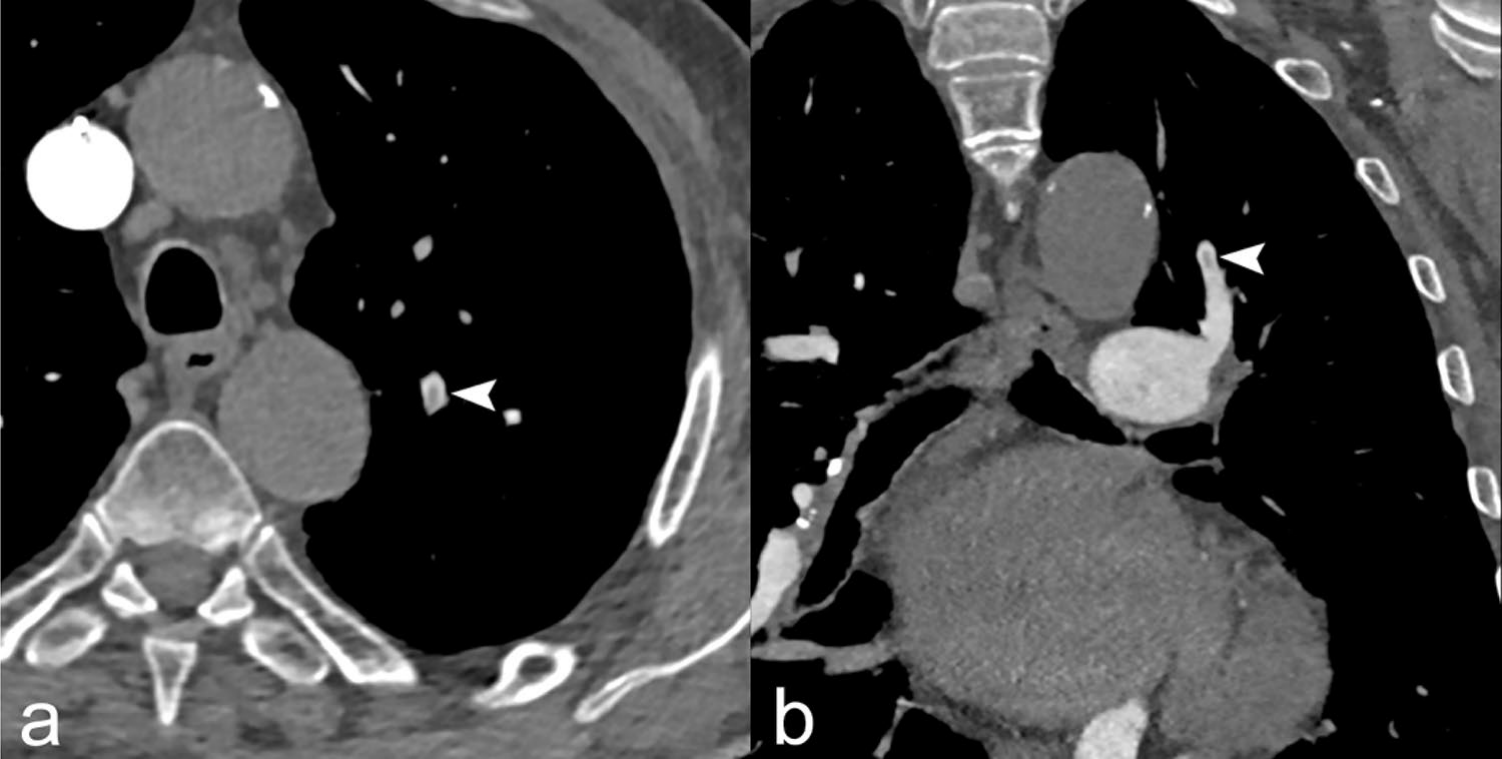
52-year-old female, axial (**a**) and coronal (**b**) CTPA images showed small and partial intraluminal filling defect of the left upper lobe segmental pulmonary artery (arrowhead)

**Fig. 4:**
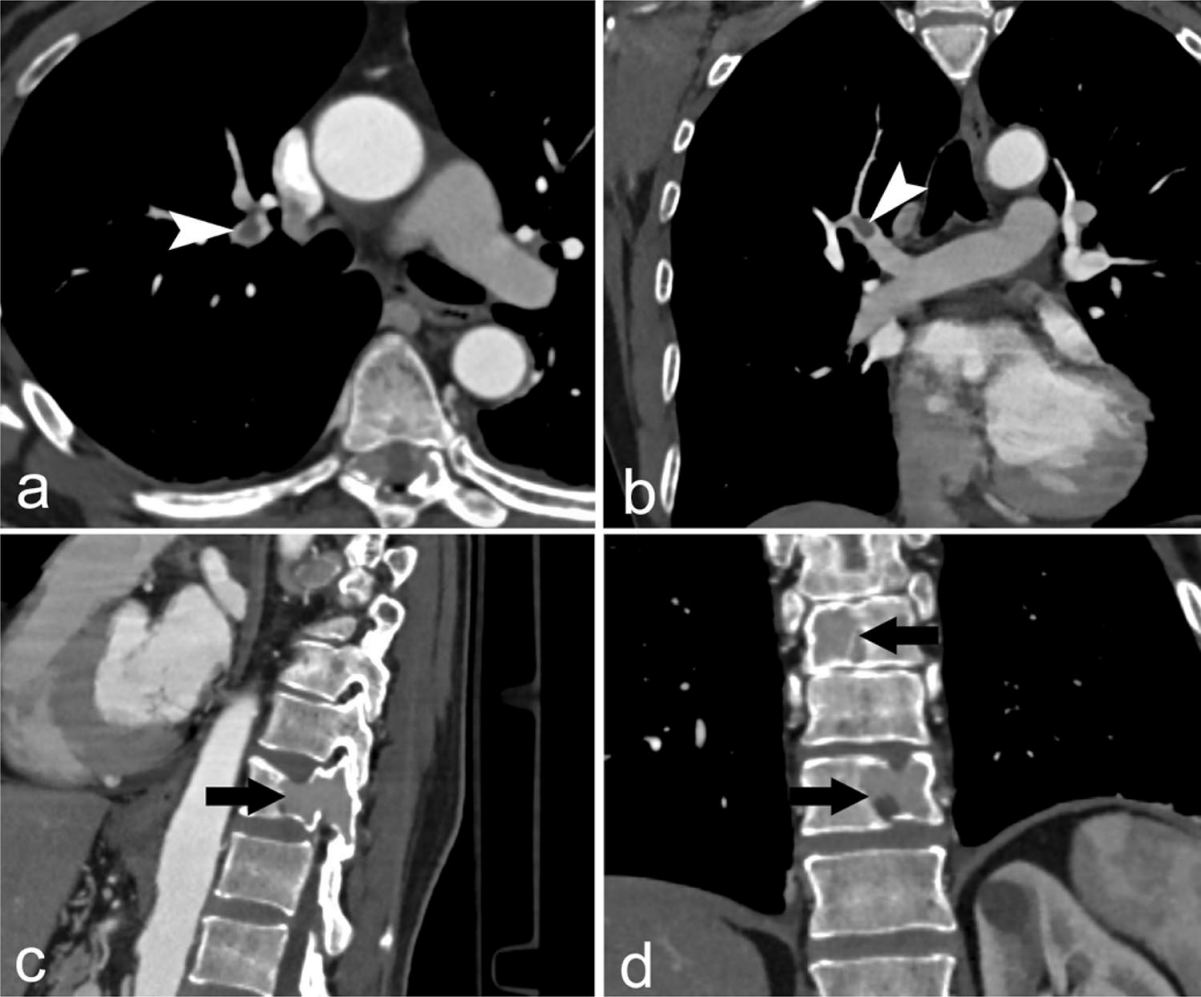
85-year-old male, axial (**a**) and coronal (**b**) CTPA images showed right upper lobe pulmonary embolus (arrowhead) but also multiple lytic bone lesions (black arrow) later diagnosed as multiple myeloma (**c**, **d**)

Of the 437 patients enrolled, PE was diagnosed in 65 patients (prevalence PE was 14.87%). Through the retrospective application of the RGS original versions, 178 patients resulted “PE likely,” while 259 patients “PE unlikely”; in the latter, we implemented D-dimer value with age-adjusted cut-off, so 43 (9.84%) CTPA requests resulted not appropriate (Fig. 2). Retrospective PE prevalence in the subgroup of patients with appropriate CTPA was 63/394 (15.99%). No thoracic change was found in 24/43 (55.81%) of patients with inappropriate CT, while 19/43 (44.19%) patients with inappropriate CTPA revealed a pulmonary disease. Namely, in this group, 2/43 (4.65%) patients, a PE was diagnosed; the first patient showed a non-opacified left upper lobe segmental pulmonary artery with a small and partial intraluminal filling defect (Fig. 3), whereas in the second case, an 85-year-old male admitted at the emergency department for low back pain, a right upper lobe pulmonary artery embolus and multiple lytic bone lesions, later diagnosed as multiple myeloma were found (Fig. 4). The alternative pulmonary diseases in this group were in 12/43 (27.81%) a parenchymal opacification (seven consolidation, two ground glass opacity), in 1/43 (2.33%), a pleural effusion, in 1/43 (2.33%) of cases, the evidence of bronchiectasis and in 1/43 (2.33%), a lung cancer was diagnosed; finally, 2/43 (4.65%) of patients showed alternative findings such as mediastinal or pleural disease (Table 3). In all these cases, a specific treatment or follow-up was necessary. The original RGS coupled with the age-adjusted D-dimer cut-off displayed a negative predictive value of 95.35% and a positive predictive value of 15.99%.

**Table 3:** CT chest findings in 43 patients with CTPA inappropriate

	*n*, %
No chest signs	24 (55.81%)
Parenchymal opacification	12 (27.91%)
PE	2 (4.65%)
Pleural effusion	1 (2.33%)
Bronchiectasis	1 (2.33%)
Lung cancer	1 (2.33%)
Alternative findings*	2 (4.65%)

*One patient with mediastinal mass, one patient with pleural disease

## Discussion

To our knowledge, this is the first study that analyzed a population applying the clinical pre-test probability from the RGS with the D-dimer level using its age-adjusted cut-offs. We selected the RGS as model to assess clinical pre-test probability, rather than the Wells rule [10]. The latter includes seven items: one of these is a subjective variable (“an alternative diagnosis less likely than PE”), thus there is an interobserver variability [11, 12].

Of the 437 patients enrolled, PE was diagnosed in 14.87% patients, and this percentage is consistent with the prevalence data reported in many studies: it varies from values of about 50% in the early 1980s studies to 5% in the latest North American one [13].

The temporal trend of the last few years shows an increase in PE diagnoses without a concomitant increase in mortality from PE; today, we have more sensitive diagnostic tools, but we must be careful with the problem of false positives and the excess treatment. The new guidelines for the diagnosis and management of acute PE aim to avoid the unnecessary imaging tests, such as CTPA that are now commonly performed in patients come to the emergency department with dyspnea and/or chest pain. CTPA is commonly used to assess patients for PE. The clinical validity of CTPA to rule out a diagnosis of PE is similar to conventional pulmonary angiography, and it has become the new gold standard for PE diagnosis [14]. Between 2000 and 2008, the use of CTPA increased by about 14 times, from 0.3 to 4% per 1000 patients, while V/Q scanning decreased by 52% [15]. Physicians request more frequently the CTPA rather than other diagnostic exams; CTPA allows formulating alternative or concomitant diagnoses, such as pleural effusion or pneumonia, able to explain the specific onset [14]. Since the untreated patients have a poor prognosis [16], many emergency physicians request an increasing number of CTPA in the fear of “missing” a PE diagnosis and receiving malpractice accusations [17]. CTPA is often required in patients who do not meet the predictive clinical criteria. In clinical practice, the systematic use of algorithms incorporating clinical prediction scores is poor [18], resulting in an overuse of the diagnostic tool with medical and economic implications. Many physicians refuse to apply clinical score models because they are supported by scientific evidence that the use of the so-called “gestalt” clinic, especially if the physician is expert, has an efficacy comparable to clinical prediction rule one [8]. Conversely, the clinical judgment is influenced by several factors, first of all the physician experience, so it lacks standardization.

In our study, the PE prevalence in the subgroup of patients with appropriate CTPA was shown to be slightly higher than the PE prevalence in the entire study population (15.99% vs. 14.87%), so the physician assessment would seem to have an accuracy comparable to the RGS coupled with D-dimer. On the contrary, our results also proved that 43/437 (9.84%) of CTPA could have been avoided by use of original RGS coupled with age-adjusted D-dimer cut-off, as it has already been demonstrated in previous experiences, namely in a prospective study of 2012. Crichlow et al. described in a smaller study population that in total 9.2 and 13.8% of CTPA procedures could have been avoided by use of PERC and Wells/D-dimer, respectively [19]. On the other hand, in our series, 19/43 (44.19%) of inappropriate CTPA resulted crucial to explain symptoms suggesting an alternative thoracic diagnosis. The possibility of findings supporting alternative diagnosis on CTPA for suspected PE and its clinical impact is well-known in the literature, and the prevalence of such incidental findings ranges from 25 to 52% in different studies [20–22]. For this reason, we still suggest an unenhanced thoracic scan before contrast agent injection for CTPA to investigate for possible alternative diagnosis especially when thoracic pain is present and vascular emergencies like aortic intramural hematoma or dissection may be found.

One confounding outcome of our experience is the fact that two PE were found in patients who received an inappropriate CTPA. In the first patient, the PE was small, partial and segmental which could explain both the “PE unlikely” as category of clinical pre-test and the lower D-dimer value. In fact, symptoms as well as biomarkers levels, may depend on the degree of vascular obstruction being small and peripheral PE often associated with a more benign clinical onset and a lower value of D-Dimer [23–25]. The second patient was an elderly male with low back pain and D-dimer level below his age-adjusted cut-off. In several studies, the use of age-adjusted D-dimer cut-off has been formally evaluated: Douma et al. [26], especially in older patients (> 70 years), and Righini et al. (2014) [6] demonstrated as the age-adjusted D-dimer cut-off is a safe and accurate tool when correctly combined with pre-test clinical probability to rule out PE and to avoid overuse thoracic imaging. In our case, the failure of the predictive model may be probably explained by an underestimation of RGS since active cancer state of the patient (multiple myeloma) was unknown when CTPA was requested.

Our analysis suffers of some limitations as it is retrospective, based on the experience of a single center and in a limited period with a consequent need of a wider validation of results with further multicentric and prospective studies.

In conclusion, despite a good prevalence of PE in our series, suggesting that in high-volume centers, the physician assessment may have a good reliability, and our retrospective analysis showed that the systematic application of the RGS coupled with age-adjusted D-dimer cut-off in patients presenting to Emergency Department with signs and symptoms of PE could lead to a better selection of patients to undergo CTPA and to a more appropriate use of this diagnostic method.
